# Brain Proteome Profiling Reveals Common and Divergent Signatures in Parkinson’s Disease, Multiple System Atrophy, and Progressive Supranuclear Palsy

**DOI:** 10.1007/s12035-024-04422-y

**Published:** 2024-08-21

**Authors:** Fiona Dick, Gard Aasmund Skulstad Johanson, Ole-Bjørn Tysnes, Guido Alves, Christian Dölle, Charalampos Tzoulis

**Affiliations:** 1 Neuro-SysMed Center of Excellence for Clinical Research in Neurological Diseases, Department of Neurology, Haukeland University Hospital, 5021 Bergen, Norway; 2 Department of Clinical Medicine, University of Bergen, Pb 7804, 5020 Bergen, Norway; 3 K.G. Jebsen Center for Translational Research in Parkinson’s Disease, University of Bergen, Pb 7804, 5020 Bergen, Norway; 4 Centre for Movement Disorders and Department of Neurology, Stavanger University Hospital, Pb 8100, 4068 Stavanger, Norway; 5 Department of Mathematics and Natural Sciences, University of Stavanger, 4062 Stavanger, Norway

**Keywords:** Proteomics, Parkinson’s disease, Multiple system atrophy, Progressive supranuclear palsy, Atypical parkinsonism, Prefrontal cortex

## Abstract

The molecular pathogenesis of degenerative parkinsonisms, including Parkinson’s disease (PD), progressive supranuclear palsy (PSP), and Multiple system atrophy (MSA), remains largely unknown. To gain novel insight into molecular processes associated with these diseases, we conducted a proteome-wide expression study in prefrontal cortex tissue from a cohort of 181 individuals, comprising PD (*N* = 73), PSP (*N* = 18), MSA (*N* = 17) and healthy control (*N* = 73). Using marker gene profiles, we first assess the cellular composition of the samples and, subsequently, identify distinct protein signatures for each disease, while correcting for cell composition. Our findings indicate that all three diseases are characterized by a structural and/or functional loss of deep cortical neurons, while PD exhibits an additional decrease in somatostatin-expressing interneurons, as well as in endothelial cells. Differential protein expression analysis identified multiple proteins and pathways with disease-specific expression, some of which have previously been associated with parkinsonism or neurodegeneration in general. Notably, we observed a strong mitochondrial signature which was present in both PD and PSP, albeit of a different composition and most pronounced in PSP, but not in MSA where immunological/inflammation-related pathways dominated. Additionally, we identified protein signatures associated with the severity of α-synuclein pathology in PD and showed that these are highly enriched in an upregulation of mitochondrial processes, specifically related to oxidative phosphorylation and in particular respiratory complexes I and IV. We identify multiple novel signatures of protein expression, associated with PD, PSP, and MSA, as well as with the severity of α-synuclein pathology in the PD brain.

**Supplementary Information:**

The online version contains supplementary material available at 10.1007/s12035-024-04422-y.

## Introduction

Neurodegenerative parkinsonisms, including Parkinson’s disease (PD), multiple system atrophy (MSA), and progressive supranuclear palsy (PSP), are severe and relentlessly progressive neurodegenerative diseases sharing the clinicopathological hallmarks of parkinsonism and degeneration of the dopaminergic neurons of the substantia nigra pars compacta (SNc). Beyond this common core, PD, MSA, and PSP are characterized by distinct clinical and pathological features [1]. Important pathological hallmarks of these syndromes are that PD is characterized by neuronal α-synuclein aggregation in the form of Lewy pathology, MSA exhibits oligodendroglial α-synuclein inclusions termed cytoplasmic glial inclusions, while PSP shows accumulation of tau in neurons and glia [1].

Despite their well-described pathology, the molecular pathogenesis of neurodegenerative parkinsonism is largely unknown. Efforts to identify pathways involved in disease initiation and progression commonly employ gene expression studies in brain tissue. However, while it is generally assumed that observed differences in mRNA levels reflect differences at the protein level, this is not always the case. The correlation between transcript and protein levels varies considerably across genes and individuals and becomes decoupled in the aging brain [2, 3]. Moreover, we have shown that the relationship between transcript and protein is further altered in the PD brain, highlighting the importance of approaching the inference of protein changes from gene expression changes with caution [4].

Few studies have assessed proteome-wide expression in the PD brain, with the majority conducted in the substantia nigra [5]. One important limitation of studying bulk substantia nigra tissue is that it typically exhibits severe neurodegeneration, with loss of approximately 80% of the dopaminergic neuronal population and extensive gliosis [6]. These alterations introduce substantial bias in differential expression analyses in bulk tissue, making it impossible to distinguish between regulatory disease-related changes and differences in underlying cell composition [7]. Additionally, any signal from surviving dopaminergic neurons is likely derived from terminal or resilient cells, thereby providing limited information about early pathogenic processes. A recent proteomics study in the PSP globus pallidus, which is severely degenerated, suffered from the same issues of cell composition bias [8].

This limitation can be partially mitigated by studying regions with milder disease involvement, such as the neocortex. However, we have shown that cell-type composition remains problematic, albeit less pronounced, even in areas considered mildly affected, such as the prefrontal cortex in PD [7, 9]. This may reflect the insufficient characterization of changes in the neocortical cell composition in neurodegenerative parkinsonisms. The few proteomics studies conducted in the PD cortex failed to address the pertinent issue of cell composition [5, 10, 11]. Moreover, to the best of our knowledge, no proteome-wide studies have been performed on cortical tissue from individuals with PSP, and only a single study has been reported in MSA [12], which did not account for cell composition.

In this work, we performed proteomics analyses in prefrontal cortex samples of a large cohort of individuals with PD (*N* = 73), PSP (*N* = 18), MSA (*N* = 17), and healthy control (*N* = 73). We first analyzed our data to comprehensively characterize the cellular composition of the samples, and subsequently identified distinct protein signatures for each disease, while correcting for cell composition.

## Results

### Estimating Sample Cell Composition from Brain Bulk-Tissue Proteomics

To estimate cell composition, we employed the marker gene profile (MGP) method for proteomics datasets, using marker genes from Kelley et. al. [13] and Velmeshev et. al. [14]. We have previously employed this approach to estimate cell composition in the striatum of PD and PSP [8]. As additional proof of concept, we assessed whether our method would recapitulate the pathology of advanced Alzheimer’s disease (AD), typically characterized by widespread cortical neuronal loss and gliosis, accompanied by tau and amyloid-beta deposition [15]. To this end, we estimated cell composition on a publicly available proteomics dataset [16] derived from postmortem frontal cortex samples of PD patients (*N* = 10), Alzheimer’s disease patients (*N* = 10), individuals exhibiting both PD and AD pathology (*N* = 10), and healthy controls (*N* = 10).

**Fig. 1:**
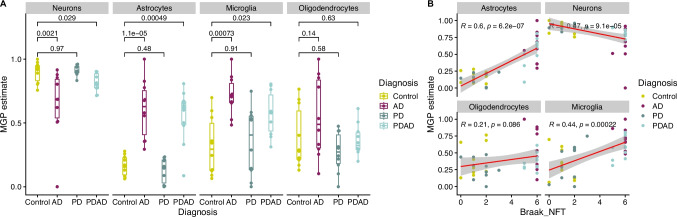
Marker gene profiles to estimate cell composition from protein expression -proof of concept in the Alzheimer’s disease brain. **A** Marker gene profile estimates (y-axis) are displayed per group (*x*-axis) and per cell type (panels). Wilcoxon test was used to calculate *p*-values. Color indicates groups. **B** Scatterplot indicating the relationship between Braak tau (*x*-axis) and marker gene profile estimates (*y*-axis). Color indicates groups. Correlation coefficient *R* was calculated using Pearson’s correlation

AD samples were indeed characterized by a significant decrease in neuronal estimates (*p* = 0.0021) and a significant increase in astrocytes (*p* = 1.1 × 10^–5^) and microglia (*p* = 0.00073), in line with what is expected by the disease pathology. Samples with PD/AD overlap pathology also exhibited a reduction in neuronal estimates (*p* = 0.029) and an increase in astrocytes (*p* = 0.00049) and microglia (*p* = 0.023), albeit to a lesser extent than in individuals with pure AD pathology (Fig. 1A). Oligodendrocyte estimates showed no significant difference between either of the groups. Furthermore, the severity of tau pathology, as measured by Braak stage [17], was positively correlated with the estimates of astrocytes (Kendall $\tau$ = 0.6 *p* = 6.2 × 10^−7^) and microglia (Kendall $\tau$ = 0.44, *p* = 0.0002), and negatively correlated with neuronal estimates (Kendall $\tau$ =  − 0.47, *p* = 9.1 × 10^−5^), recapitulating the known correlation between neuronal loss, gliosis, and tau pathology [18] (Fig. 1B). We thus concluded that the method as well as the selected marker “genes” were appropriate for the use on proteomics data.

### Proteomics-Based Estimation of Cell Composition in the Prefrontal Cortex (PFC) of PD, PSP, and MSA

**Fig. 2:**
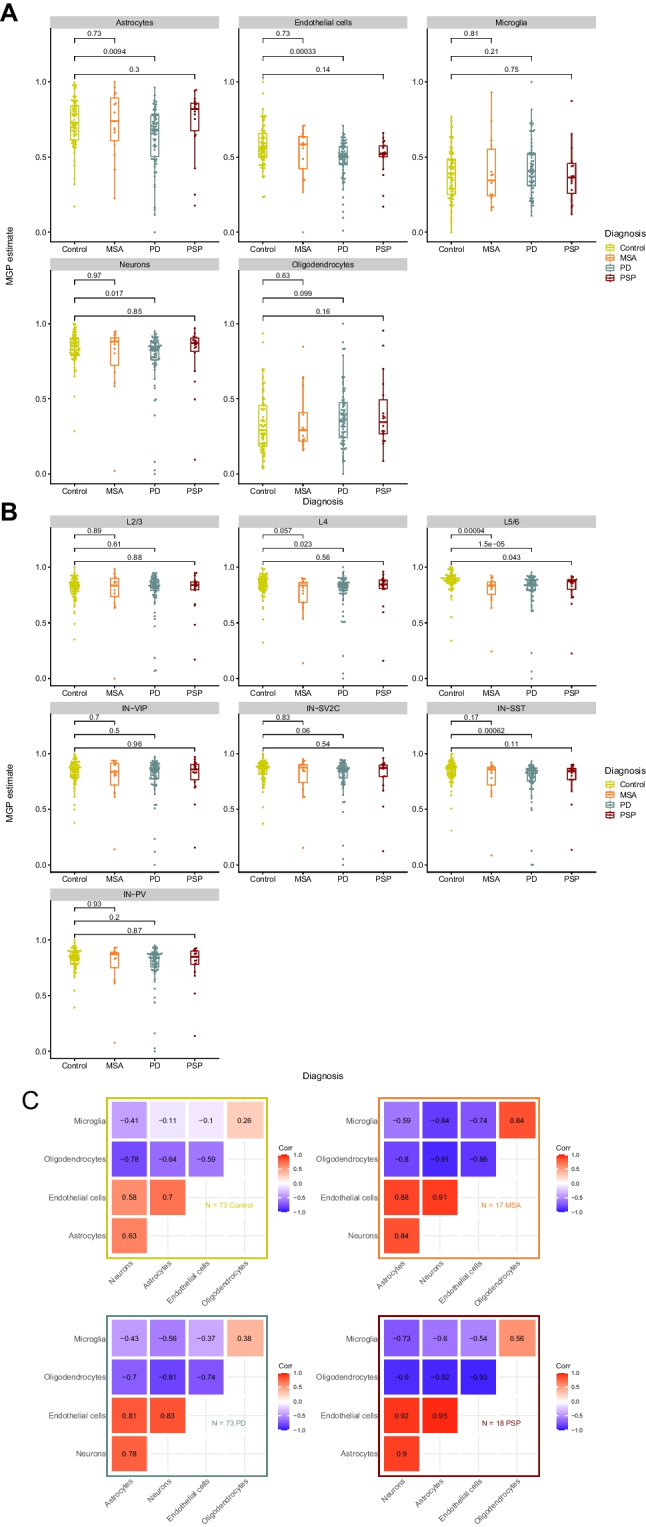
Estimates of cell composition in the prefrontal cortex of PD, PSP, and MSA**. ** Cell estimates (*y*-axis) based on marker gene profiles per group (*x*-axis, color coding) and cell type (panels). Wilcoxon tests were used to compare the control group (yellow) to the disease group. **A** Major cell types **B** Neuronal subtypes **C** Heatmaps display pairwise correlations of cell estimates between cell types per group. Color indicates positive (red) and negative (blue) correlation. Color intensity indicates strength of correlation

Next, we estimated the MGPs for neurons, oligodendrocytes, microglia, astrocytes, and endothelial cells in our samples (Fig. 2A). Compared to controls, PD exhibited a significant decrease in neuronal estimates (*p* = 0.017), a decrease in astrocyte estimates (*p* = 0.01), and a highly significant decrease for endothelial cell estimates (*p* = 0.0003). No change was observed in estimates of oligodendrocytes and microglia. MSA and PSP exhibited no significant difference from controls for any of the cell types.

Furthermore, we estimated the MGPs for neuronal subtypes, for which marker genes have been defined [13, 14]. In PD, changes in neuronal estimates followed an anatomical gradient, with superficial cortical layers showing mild or no significant changes compared to controls (Fig. 2B). No change was observed for neuronal subtypes in layers 2 and 3. In layer 4 excitatory neuronal estimates were significantly decreased in PD (L4, *p* = 0.023), while inhibitory neurons (IN-SV2C and IN-VIP) showed no difference. A more substantial decrease was observed in layers 5 and 6 for both corticofugal projection neurons (L5/6, *p* = 1.5 × 10^−5^) and inhibitory somatostatin interneurons (IN-SST, *p* = 0.0006). The only exception to this trend was inhibitory parvalbumin interneurons (IN-PV, layer 6) which did not show a significant difference. In PSP, the only significant difference was observed for corticofugal projection neurons in layers 5 and 6 (*p* = 0.043). MSA samples showed a trend for a decrease in excitatory neuronal estimates in layer 4 (L4, *p* = 0.057) and a significant decrease for excitatory neurons in layers 5 and 6 (L5/6, *p* = 0.0009). Using pairwise (Pearson) correlation between cell type estimates, we found that neuronal estimates positively correlated with those of both astrocytes and endothelial cells and negatively correlated with microglia and oligodendrocytes (Fig. 2C).

### PD, PSP, and MSA Exhibit Partially Overlapping Differential Protein Expression Signatures

Prior to differential protein expression (DPE) analysis, we performed surrogate variable analysis to identify sources of bias in the data. After iteratively adding variables (age, sex, cell type estimates, and batch) to the base model and correlating surrogate variables with remaining features, we concluded with the following model design for the DPE analysis: “expression ~ age + sex + batch + endothelials + neurons + diagnosis”. In a final round of surrogate variable analysis, we found no high correlation (|*r*|< 0.2) between the first surrogate variable and any other variable. This model design is in line with the cell-estimate differences we reported above, where we found the biggest differences in cell composition between cases and controls for neurons and endothelial cells.

In PD, we identified *N* = 714 significantly (False discovery rate (FDR) < 0.05) differentially expressed proteins, comprising *N* = 371 upregulated and *N* = 343 downregulated proteins. The top 20 significantly differentially expressed proteins by fold change are shown in Table 1, and a full account of all differentially expressed proteins is given in Table S2.

**Table 1:** Top 20 differentially expressed proteins

Gene symbol	Protein accession	Log fold-change	*p*-value	Adjusted *p*-value
PD versus control				
KRT9	P35527	0.424	1.57 × 10^−03^	1.58 × 10^−02^
CHRM3	P20309	0.417	8.93 × 10^−14^	1.85 × 10^−10^
KRT5	P13647	0.393	1.84 × 10^−03^	1.76 × 10^−02^
KRT10	P13645	0.385	4.02 × 10^−03^	2.98 × 10^−02^
KRT1	P04264	0.384	3.57 × 10^−03^	2.75 × 10^−02^
KRT2	P35908	0.367	4.09 × 10^−03^	3.01 × 10^−02^
SYT2	Q8N9I0	0.359	8.27 × 10^−06^	4.14 × 10^−0^4
SLC17A6	Q9P2U8	0.329	1.09 × 10^−09^	5.01 × 10^−07^
CDIP1	Q9H305	− 0.316	3.09 × 10^−06^	2.18 × 10^−04^
CHGA	P10645	0.314	9.82 × 10^−13^	1.36 × 10^−09^
VGF	O15240	− 0.31	7.03 × 10^−08^	1.12 × 10^−05^
CLDN11	O75508	− 0.307	4.84 × 10^−03^	3.41 × 10^−02^
RTN1	Q16799-3	− 0.299	5.23 × 10^−07^	5.72 × 10^−05^
GPR37L1	O60883	− 0.29	8.17 × 10^−05^	2.20 × 10^−03^
ARG2	P78540	0.28	3.44 × 10^−05^	1.18 × 10^−03^
SCN4B	Q8IWT1	0.277	5.02 × 10^−05^	1.57 × 10^−03^
CLDN10	P78369	0.269	4.13 × 10^−05^	1.35 × 10^−03^
GM2A	P17900	− 0.267	9.17 × 10^−09^	2.38 × 10^−06^
S100A12	P80511	0.261	6.69 × 10^−03^	4.21 × 10^−02^
CD9	P21926	− 0.252	1.80 × 10^−03^	1.72 × 10^−02^
PSP versus control				
FTL	P02792	0.476	1.26 × 10^−06^	1.74 × 10^−03^
CD200	P41217	− 0.456	2.05 × 10^−03^	4.76 × 10^−02^
RLBP1	P12271	0.408	1.25 × 10^−03^	3.70 × 10^−02^
ENTPD2	Q9Y5L3	0.407	2.23 × 10^−03^	4.95 × 10^−02^
CDC42EP4	Q9H3Q1	0.374	6.64 × 10^−04^	3.00 × 10^−02^
DTNA	Q9Y4J8-11	0.371	1.24 × 10^−03^	3.70 × 10^−02^
CP	P00450	− 0.365	5.17 × 10^−04^	2.87 × 10^−02^
A1BG	P04217	− 0.357	7.49 × 10^−04^	3.12 × 10^−02^
LZIC	Q8WZA0	0.349	3.29 × 10^−04^	2.68 × 10^−02^
CDIP1	Q9H305	− 0.345	6.94 × 10^−04^	3.00 × 10^−02^
CLDN10	P78369	0.317	1.36 × 10^−03^	3.88 × 10^−02^
NPTX2	P47972	− 0.317	4.52 × 10^−04^	2.81 × 10^−02^
ATP2B4	P23634-6	0.303	1.22 × 10^−06^	1.74 × 10^−03^
VWF	P04275	− 0.292	1.18 × 10^−03^	3.64 × 10^−02^
TF	P02787	− 0.292	3.57 × 10^−0^5	7.96 × 10^−03^
PSAP	P07602-3	− 0.27	2.23 × 10^−04^	2.11 × 10^−02^
NECAP2	Q9NVZ3	0.262	1.96 × 10^−03^	4.70 × 10^−02^
GNG5	P63218	0.257	5.08 × 10^−04^	2.85 × 10^−02^
HEPACAM	Q14CZ8	0.25	6.75 × 10^−04^	3.00 × 10^−02^
ARF3	P61204	0.231	3.68 × 10^−04^	2.68 × 10^−02^
MSA versus control				
AQP4	P55087	0.526	8.84 × 10^−05^	2.45 × 10^−02^
TCEAL5	Q5H9L2	0.399	4.58 × 10^−04^	3.68 × 10^−02^
ATOX1	O00244	0.357	7.90 × 10^−04^	4.97 × 10^−02^
SARNP	P82979	0.333	1.45 × 10^−04^	3.01 × 10^−02^
JPT1	Q9UK76	0.309	7.34 × 10^−05^	2.35 × 10^−02^
ADCK1	Q86TW2	− 0.3	3.03 × 10^−05^	2.35 × 10^−02^
CDC5L	Q99459	0.298	2.39 × 10^−05^	2.35 × 10^−02^
GM2A	P17900	− 0.292	7.25 × 10^−05^	2.35 × 10^−02^
SLC6A11	P48066	0.289	1.67 × 10^−04^	3.03 × 10^−02^
PCNP	Q8WW12	0.258	2.26 × 10^−05^	2.35 × 10^−02^
DR1	Q01658	0.25	2.96 × 10^−04^	3.14 × 10^−02^
KCTD8	Q6ZWB6	0.244	3.60 × 10^−04^	3.40 × 10^−02^
SERBP1	Q8NC51	0.237	2.23 × 10^−04^	3.13 × 10^−02^
MAP4	P27816-3	0.231	4.56 × 10^−04^	3.68 × 10^−02^
TOM1	O60784-2	0.225	5.56 × 10^−05^	2.35 × 10^−02^
GPT	P24298	0.222	4.23 × 10^−04^	3.68 × 10^−02^
CNN3	Q15417	0.221	1.06 × 10^−04^	2.74 × 10^−02^
SYNPO	Q8N3V7	0.211	3.02 × 10^−04^	3.14 × 10^−02^
ZFR	Q96KR1	0.2	2.91 × 10^−04^	3.14 × 10^−02^
DDT	P30046	0.199	5.67 × 10^−04^	4.21 × 10^−02^

Differentially expressed proteins (adjusted *p*-value < 0.05) were sorted by absolute log fold-change to select the top 20 from each analysis. *p*-values were adjusted during differential protein expression analysis according to the Benjamin-Hochberg method

Gene-set enrichment analysis revealed 27 significantly differentially expressed pathways, most of which were related to mitochondrial function and the proteasome. Specifically, proteasomal subunits (including PSM-A-E), subunits of the mitochondrial respiratory chain (MRC) complex V, and proteins of the mitochondrial small ribosomal subunit were together driving the enrichment of about 20 of the significant pathways (Fig. S2A). The mitochondrial ribosomal pathways were downregulated, while the proteasomal pathways and complex V subunits were upregulated. The remaining significant pathways included an upregulation of calcium signaling, the lysosome, and unfolded protein response.

In PSP, we identified *N* = 187 significantly (FDR < 0.05) differentially expressed proteins, comprising *N* = 112 upregulated and *N* = 75 downregulated (Table 1, Table S2). Gene-set enrichment analysis revealed *N* = 30 significantly differentially expressed pathways, which were primarily driven by the downregulation of subunits of the mitochondrial ribosome, and of the MRC complexes I and V (Fig. S2B). The remaining significant pathways included the downregulation of the lysosome and the upregulation of the spliceosome (Table S2).

In MSA, we identified *N* = 66 significantly (FDR < 0.05) differentially expressed proteins, comprising *N* = 21 downregulated and *N* = 45 upregulated (Table 1, Table S2). Gene-set enrichment analyses identified *N* = 79 significantly differentially expressed pathways, showing substantial biological diversity and including an upregulation of the spliceosome, and downregulation of chemokine signaling, the melanosome, and the leukocyte trans-endothelial migration pathway, as the top pathways (ranked by normalized enrichment score). We noted that two proteins, Ras-related C3 botulinum toxin substrate 1 (RAC1) and cell division control protein 42 (CDC42), were at the leading edge of more than 16 of the significantly differentially expressed pathways (Fig. S2C). These pathways, however, did not relate to one specific biological function but were biologically diverse, including “chemokine signaling,” “axon guidance,” “regulation of cell shape,” and “phagocytosis.” RAC1 and CDC42 are both ubiquitously expressed small guanosine triphosphate hydrolases (GTPases), involved in a broad spectrum of cellular functions. Interestingly, RAC1 has been implicated in the regulation of α-synuclein-induced toxicity in a Caenorhabditis elegans (C. elegans) model [19], while CDC42 plays a role in the regulation of senescence [20].

**Fig. 3:**
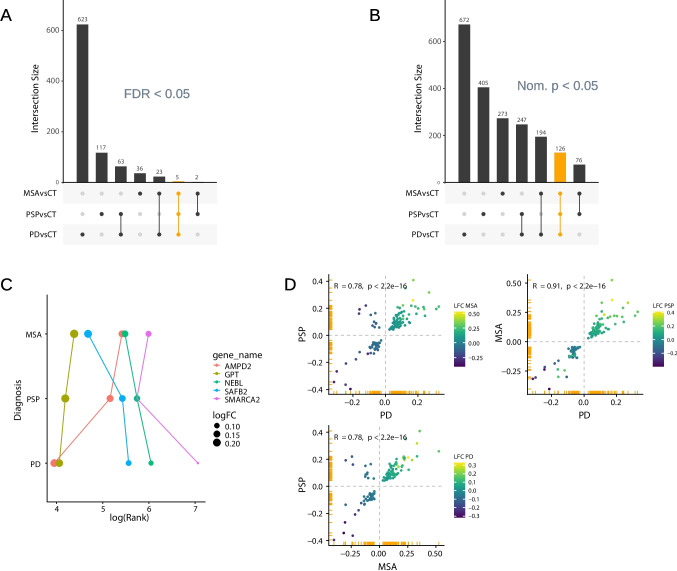
Overlap of differentially expressed proteins across diseases. Intersection sets (*x*-axis) of differentially expressed proteins (DEP) significant at **A** FDR 0.05 and at **B** nominal significance level from each analysis (PD versus control, PSP versus control, and MSA versus control) are sorted by set size (*y*-axis). Orange color indicates the intersection set of DEP common to the three analyses. **C** DEP common to all three diseases (at FDR < 0.05) are arranged by their rank (log-scaled, *x*-axis) in the respective analysis (*y*-axis). Rank was calculated by sorting DEP by absolute log fold change. Color indicates the protein, and point size indicates log fold change. **D** Comparison of log fold change (*x* and *y* axes) of DEP common to all three diseases (at nominal *p* < 0.05) between analyses. Data points in the first and third quadrants represent DEP with agreeing direction of change (between disease and control). Color indicates log fold change of the analysis not displayed on either *x* or *y* axis. Correlation coefficient was calculated using Pearson’s correlation

Finally, we investigated the similarities across the diseases by assessing the overlap of differentially expressed proteins both at the FDR level, i.e., the intersection of proteins with FDR < 0.05 in all disease group analyses (Fig. 3A), and the nominal significance level, i.e., the intersection of proteins with nominal *p*-value < 0.05 in all disease group analyses (Fig. 3B). We identified *N* = 5 common differentially expressed proteins at FDR < 0.05, all of which were upregulated: AMPD2, GPT, NEBL, SAFB2, and SMARCA2 (Fig. 3C). Due to the lower number of samples in the MSA and PSP groups, and thereby a possibly reduced power in these analyses, we preferred not to draw any conclusions from the observed variance in effect size. At the nominal significance level, we identified *N* = 126 common proteins (Table S2) across all three diseases. These did not show any significant enrichment in over-representation analysis. Finally, we investigated the log-fold change correlation between the groups among the *N* = 126 common proteins. The highest correlation of fold change was seen between PD and MSA, where all overlapping proteins changed in the same direction (Fig. 3D). While a strong correlation was also seen between PD and PSP, we identified 11 proteins (SLC35A4, FTH1, MICOS10, SAR1B, RAB2A, CDC42, RHOA, GNA13, RAB35, YKT6, VPS13C) upregulated in PSP but downregulated in PD (and in MSA). These proteins were enriched for processes related to nucleotide binding, guanosine diphosphate (GDP) binding, or GTPase activity. In the cellular component ontology, the significant pathways were all related to the Golgi apparatus.

### Top Features Discriminating PD Samples from Controls

**Fig. 4:**
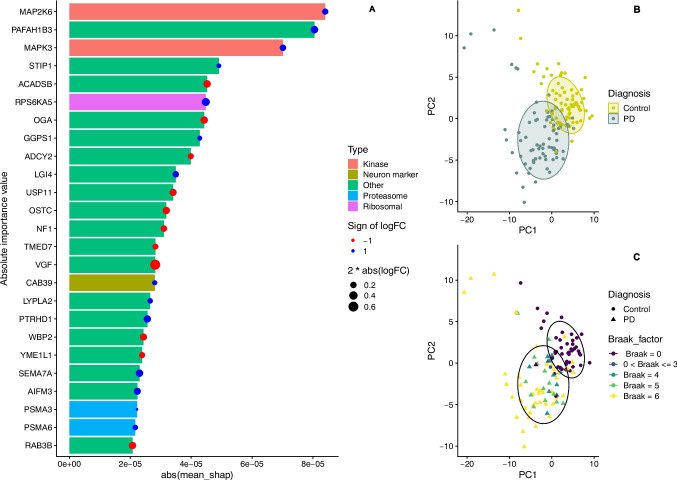
Predictive features in PD versus control classification**. A** The top *N* = 25 proteins (*y*-axis) are sorted by their importance value (*x*-axis). Bar color reflects manually categorized biological function. Data point color indicates up- (blue) and down- (red) regulation in PD (based on differential expression analysis). Data point size reflects magnitude of fold change. **B, C** PD and control samples are displayed in the first and second principle component space based on the expression values of all DEP with non-zero importance value. Color in B indicates diagnosis, and color in C indicates Braak α-synuclein staging score

To further characterize the differentially expressed protein signature of the disease, we employed an ensemble learning methodology and trained a model to predict whether a sample belonged to the PD or control group. Through this approach, we ranked proteins based on their ability to separate PD from controls. Due to the low number of samples and imbalance between control and disease samples in PSP and MSA, we limited this analysis to the PD *versus* control comparison. We were able to retrieve an importance measure for each protein reflecting the level of its contribution to the separation of PD and control samples. Using this method, we identified *N* = 157 proteins with non-zero importance values, of which the top 25 (i.e., the 25 with the highest contribution to the separation of PD and control samples) are displayed in Fig. 4A, while a full list is provided in Supplementary Table S3. These proteins were most important in the prediction of the condition variable, i.e., they separated PD from controls in our data. Principal component (PC) analysis based on the expression values of the 157 proteins showed that PD and control samples were separated in the first (PC1) and second (PC2) principal component space (Fig. 4B and C). Additionally, PC1 was significantly associated with the condition variable (i.e., PD or control, *p* < 2.2 × 10^−16^). Furthermore, we observed a significant association between PC1 and Braak α-synuclein stage (*p* < 2.2 × 10^−16^), suggesting that the expression profile of these protein features is associated with the severity of α-synuclein pathology.

Interestingly, several of the top 25 proteins (Fig. 4A) had a known link to parkinsonism and/or broader neurodegeneration and aging. Among the downregulated ones, we noted RAB3, a GTPase protein involved in synaptic vesicle transport, which has been shown to be protective in neuronal models of α-synucleinopathy [21], the neurosecretory protein VGF (nonacronymic), involved in the catecholamine secretory pathway [22] and found to be decreased in cerebrospinal fluid (CSF) from individuals with PD [23], the oligosaccharyltransferase OSTC (also known as DC2), which is involved in the processing of amyloid precursor protein (APP) [24], and OGA, a glycoside hydrolase shown to be protective against α-synuclein aggregation in vitro [25]. Notable examples among the upregulated proteins included the proteasomal subunits PSMA3 and PSMA6, mitochondrial apoptosis-inducing factor AIFM3 and PTRHD1, a protein with a possible role in the ubiquitin-proteasome system, and loss of function mutations that cause juvenile-onset parkinsonism [26].

### The Severity of α-synuclein Pathology is Associated with Upregulation of Mitochondrial Pathways

**Fig. 5:**
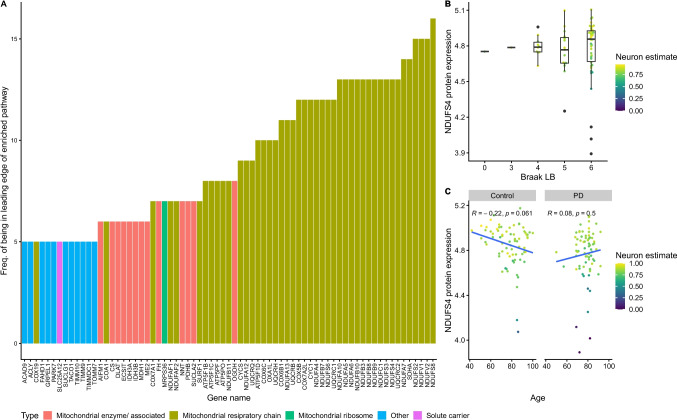
Protein expression associated with the severity of α-synuclein pathology (Braak stage) in PD. **A** Proteins in the leading edge of more than 3 significant pathways were arranged on the x-axis by their frequency of occurrence (*y*-axis). Color indicates the type of protein. **B** Boxplots showing the distribution of NDUFS4 protein expression (*y*-axis) per Braak alpha-synuclein staging score (*x*-axis), for all samples for which the Braak score was available. Color indicates neuronal estimates. **C** Scatterplot displaying the relationship between age (*x*-axis) and NDUFS expression (*y*-axis) per group (panels). Color indicates neuronal estimates

We performed differential expression analysis to identify proteins associated with the severity of disease pathology. Since pathology staging was not available for our MSA cases and was only available for five PSP cases, this analysis was limited to PD. We identified differential protein expression associated with the severity of α-synuclein pathology, as measured by the corresponding Braak staging scores in the *N* = 69 PD samples for which this data was available. We identified *N* = 35 proteins significantly (FDR < 0.05) associated with Braak stage for α-synuclein, of which *N* = 7 were downregulated and *N* = 28 were upregulated. Gene set enrichment analysis revealed in *N* = 45 significant pathways (Table S2), most of which were related to mitochondrial function. Specifically, the higher Braak stage was associated with an upregulation of processes related to oxidative phosphorylation, including complexes I and IV of the MRC. Examining the frequency of the significant proteins’ membership in enriched pathways, we found that nuclear-encoded complex I subunits were driving the enrichment of over 13 significant pathways. Furthermore, the leading edge of more than 5 significant pathways was related to mitochondrial function (Fig. 5A).

Unlike its downregulation in PD compared to controls, the mitochondrial ribosome was upregulated with increasing Braak score within the PD group. We investigated this further by selecting all mitochondrial ribosomal proteins (MRP) with nominal significant *p*-values (*N* = 6 with nom. *p* < 0.05) and adjusted these using the Bonferroni multiple testing correction. Of these, we identified five as significant, all upregulated: *MRPS26*, *MRPL17*, *MRPL15*, *MRPL20*, and *MRPL12.* Thus, the ribosomal upregulation with increasing Braak stages involved proteins of both the small and large ribosomal subunit.

To further investigate the observed upregulation of mitochondrial pathways, we selected a nuclear-encoded complex I subunit (NDUFS4) which has been extensively studied in the PD brain and shown to be representative of changes in all modules of the complex [27]. As suggested by the enrichment analysis, PD samples showed an increase in expression of NDUFS4 with increasing Braak stage (Fig. 5B), with the exception of a few outlier samples which had very low neuronal estimates, likely explaining the low complex I expression. In line with the known decline in mitochondrial function which occurs with aging [28, 29], NDUFS4 levels declined with increasing age in controls. However, this behavior was not present in PD, where NDUFS4 levels did not change with age (Fig. 5C), likely due to the α-synuclein pathology-associated upregulation. In Supplementary Figs. S3 and S4, we provide a detailed overview of the log-fold changes of subunits of the mitochondrial ribosome and the MRC from both the PD versus controls and the Braak analysis.

## Discussion

We characterize proteome-wide expression profiles in the prefrontal cortex of three neurodegenerative parkinsonisms: PD, MSA, and PSP. Our approach, employing proteomics-derived estimates of cellularity, suggests that the prefrontal cortex harbors altered cell composition in these diseases, which must be accounted for in order to correctly interpret bulk-proteomics data. While the prefrontal cortex is generally reported to exhibit mild neurodegeneration in these disorders [1], it is important to consider that this assessment is based primarily on the distribution and extent of the proteinopathy (i.e., α-synuclein or tau), rather than measurements of the actual cell composition of the tissue. The latter has not been adequately studied, despite the fact that clinical signs of frontal lobe dysfunction, such as executive function deficits, occur in all three disorders [30–32].

In PD, we find decreased neuronal estimates and increased astrocyte estimates, which is in line with previously reported transcriptome-based estimates [9]. These findings may reflect a lower neuronal content and/or cellular dysfunction with decreased neuronal and increased astrocytic transcription/translation. Interestingly, the most significant neuronal decrease in PD was seen for the estimates of inhibitory somatostatin-expressing neurons. Previous studies have shown reduced levels of somatostatin in the PD frontal cortex and CSF [33, 34]. Seen together with those previous reports, our findings suggest that there may be a selective dysfunction and/or degeneration of somatostatin-expressing neurons in the PD cortex. This warrants validation by histological studies.

The most prominent cell composition difference between PD samples and controls was a significant decrease in the estimates of endothelial cells. This may reflect microvascular regression/degeneration and/or microvascular dysfunction. Evidence for microvascular changes, including vascular regression, blood-brain barrier disruption, and cerebral blood flow abnormalities have been described in PD [35], but their nature and role remain controversial. Our findings strengthen the notion that vascular pathology, specifically in the form of vascular regression may occur in PD.

All three diseases, MSA, PSP, and PD, exhibited a selective decrease in the estimates of deep cortical neurons located in layers 5–6. Pyramidal neurons from these layers are a major source of output from the neocortex to other cortical and subcortical areas, including the thalamus, striatum, brainstem, and spinal cord [36]. Dysfunction and degeneration of these neurons could, therefore, contribute to both motor and non-motor impairment in these diseases. Further research is warranted to elucidate which exact neuronal types are affected in these layers, as well as the reasons for this selective vulnerability.

Case-control comparisons revealed numerous significant differentially expressed proteins. While some of these overlapped across diseases, the vast majority were disease-specific. Some of the top findings per disease will be discussed here in light of current knowledge. It should be stressed, however, that this is not meant to be an exhaustive discussion of the results, but rather one of unexpected findings, as well as findings with a known link to parkinsonism and/or broader neurodegeneration.

The top differentially expressed proteins in PD by fold change comprised an upregulation of multiple members of the keratin gene family (KRT-1, 2, 5, 9, and 10). While most keratins are abundantly expressed in the skin, hair, nails, and tongue, several are expressed across multiple tissues, including the brain. Based on the Genotype-Tissue Expression (GTEx) project, all of KRT-1, 2, 5, 9, and 10 are expressed in the brain, including the frontal cortex, with KRT-10 being most highly expressed in this region [37]. Interestingly, increased levels of KRT-9 have been found in the CSF from patients with Alzheimer’s disease, multiple sclerosis, and neuromyelitis optica compared to controls, and it has been proposed that this may be due to leakiness of the blood-brain barrier [38]. The role of altered keratin expression in neurodegeneration is, however, poorly understood.

In addition, PD exhibited an upregulation of muscarinic cholinergic receptor 3 (CHRM3). This may represent denervation hypersensitivity caused by the loss of cholinergic input to the prefrontal cortex from the basal forebrain nuclei [1]. This finding corroborates a previous positron emission tomography (PET) study showing increased ligand binding for muscarinic cholinergic receptors in the PD, but not the PSP brain [39].

Other notable findings included the upregulation of two proteins of the granin neuropeptide family, VGF and chromogranin A (CHGA). These proteins are essential for catecholaminergic metabolism and transmission [22]. Moreover, VGF has been shown to play a role in regulating synaptogenesis and neurogenesis, learning, and memory [40]. In line with our findings, both CHGA and VGF were found to be decreased in CSF from individuals with PD [23] and AD [40]. It is likely that the loss of CHGA and VGF is the result of the widespread catecholaminergic deficit characterizing PD [41].

Of interest was also the downregulation of CD38, a cell surface glycoprotein with a key role in nicotinamide adenine dinucleotide (NAD) metabolism and immune regulation. CD38 consumes intracellular NAD^+^, lowering its levels, and generates cyclic adenosine diphosphate ribose (cADPR), an important signaling molecule for calcium homeostasis [42]. Aberrant NAD metabolism has been linked to PD [43, 44]. It is possible that the CD38 downregulation is a compensatory effort to decrease NAD consumption. Alternatively, this may contribute to cell dysfunction via dysregulation of calcium metabolism.

Notable single protein changes in PSP included downregulation of CD200, which plays a key role in protecting neurons from microglia-induced neurotoxicity [45], and of the synaptic protein neuronal pentraxin-2 (NPTX2), which has been found to be decreased in CSF of patients with PD, PSP, and MSA, and likely reflects synaptic dysfunction and loss in the prefrontal cortex [46]. This protein was also significantly decreased in our PD samples, but not in the MSA samples. Additionally, we noted altered expression in ceruloplasmin, and ferritin light chain (FTL) involved in copper and iron metabolism, respectively. Ceruloplasmin mutations cause Wilson’s disease, while FTL mutations cause neuroferritinopathy, both of which are characterized by basal ganglia degeneration and severe movement disorders, including parkinsonism [47, 48].

Top differentially expressed proteins in MSA, included a potent upregulation of aquaporin 4 (AQP4), an astrocytic protein integral to the glymphatic system, which contributes to the clearance of amyloid-β and has been proposed to play a role in the clearance of α-synuclein [49]. In addition, we noted an upregulation of the cytoplasmic copper chaperone ATOX1, which has been found to inhibit α-synuclein aggregation in vitro [50].

At the pathway level, there was a robust mitochondrial signal in PD and PSP but not in MSA. PD was characterized by downregulation of the mitochondrial ribosome and upregulation of nuclear-encoded subunits of complex V, while no change was seen for the electron transferring complexes (I–IV). These findings do not align with immunohistochemical studies of the PD prefrontal cortex, which show a mosaic distribution of neuronal complex I deficiency [51]. However, a direct comparison of results from immunohistochemistry and proteomics is not straightforward, as the first commonly assesses individual neuronal bodies, while the latter measures differences in homogenized bulk tissue, without cell-specific resolution, and including proteins from neuronal processes and synapses. Compared to PD, PSP had a more pronounced mitochondrial signature with downregulation of both the mitochondrial ribosome and the MRC, including complexes I and V. While mitochondrial pathology is an established feature of PD [52], this is much less studied in PSP. Interestingly, our findings suggest there may be a stronger mitochondrial component in PSP than in PD. A similar trend was reported in bulk tissue proteomics from the globus pallidus [53], although those findings did not survive correction for cell-type composition [8].

Within the PD group, the levels of oxidative phosphorylation (OXPHOS) proteins and the mitochondrial ribosome changed congruently and were positively correlated with increased Braak staging. Interestingly, it has been shown that late α-synuclein pathology (i.e., formed pale bodies and Lewy bodies) preferentially occurs in neurons with quantitatively intact respiratory chain, while early α-synuclein pathology has a strong predilection for complex I deficient neurons [51, 54, 55]. This suggests that the formation of mature α-synuclein pathology requires intact mitochondrial respiration and that respiratory deficient neurons harboring starting α-synuclein pathology are less likely to survive. Thus, the observed upregulation of the MRC may represent a response to a need for higher bioenergetic efficiency to promote neuronal survival in advanced stages of PD and widespread α-synuclein pathology.

In conclusion, our findings reveal evidence of altered cell composition, as well as multiple novel differential protein expression signatures in the prefrontal cortex of individuals with PD, PSP, and MSA. Our study has several limitations. Bulk tissue proteomics has low sensitivity and is generally biased towards abundantly expressed proteins. Therefore, despite a large sample size, we cannot exclude changes in proteins of low abundance and related pathways. The estimates of cell composition are based on the expression of protein markers, not actual cell counts. Therefore, while our findings are consistent with altered cell composition in the tissue, this must be confirmed with systematic histological studies. Finally, while we are adjusting for differences in cell composition between the groups, the bias of cell composition cannot be completely removed from bulk tissue studies. Thus, we cannot exclude the possibility that this bias may still contribute to some of our findings.

## Methods

### Data and Code Availability

The datasets supporting the conclusions of this article are included within the article and its supplementary files. The source code including the description and all data for the analyses is available on GitHub: https://github.com/fifdick/DPE_parkinsonisms_brain. Any additional information required to reanalyze the data reported in this paper is available from the lead contact upon request.

### Cohorts

All experiments were conducted in fresh-frozen prefrontal cortex (Brodmann area 9) tissue from a total of 181 individuals comprising PD patients (*N* = 73, age 78.2 ± 7.21 years), MSA patients (*N* = 17, age 66.6 ± 6.93), PSP patients (*N* = 18, age 75.5 ± 8.16 years), and neurologically healthy controls (*N* = 73, age 77.6 ± 12.8 years). Controls had no known neurological disease and were matched for age and sex. All cases were confirmed neuropathologically, whereas controls had no pathological evidence of neurodegeneration. Cohort demographics including sex and age of all individuals are listed in Table S1.

Ethical permission for these studies was obtained from our regional ethics committee (REK 2017/2082, 2010/1700, 131/04). Written formal informed consent was obtained from all participants or their next of kin.

### Mass Spectrometry Sample Preparation

Briefly, brain samples were lysed using between 30 uL of lysis buffer (consisting of 6 M Guanidinium Hydrochloride, 10 mM (tris(2-carboxyethyl)phosphine) TCEP, 40 mM chloroacetamide (CAA), 50 mM 4-(2-hydroxyethyl)-1-piperazineethanesulfonic acid (HEPES) pH8.5). Samples were placed in the Barocycler 2320EXT (Pressure BioSciences) and lysed by 60 cycles of 50 s 45000 psi and 10 s atmospheric pressure at 33 oC. The samples were spun for 10 min at 14000xg and the protein content of the supernatant was determined by bicinchoninic acid assay (BCA). Twenty micrograms of the sample was diluted to 20 µL with lysis buffer and taken forward for digestion. Samples were diluted 1:3 with digestion buffer (10% acetonitrile, 50 mM HEPES, and pH 8.5), endoproteinase LysC (Mass Spec (MS) grade, Wako) was added in a 1:50 (enzyme to protein) ratio, and samples were incubated at 37 °C for 4 h. Samples were further diluted to a final 1:10 with digestion buffer and trypsin (MS grade, Sigma) was added in a 1:100 (enzyme to protein) ratio after which samples were incubated overnight at 37 ℃. Samples were acidified by adding 2% trifluoroacetic acid (TFA) to a final concentration of 1%. Prior to tandem mass tag (TMT) labeling, the peptides were desalted on a SOLAµ solid phase extraction (SPE) plate (horseradish peroxidase (HRP), Thermo). Between each application, the solvent was spun through by centrifugation at 1500 revolutions per minute (RPM). For each sample, the filters were activated with 200 ul of 100% methanol, then 200 ul of 80% acetonitrile, and 0.1% formic acid. The filters were subsequently equilibrated 2 × with 200ul of 1% TFA and 3% acetonitrile, after which the sample was loaded. After washing the tips twice with 200 ul of 0.1% formic acid, the peptides were eluted into clean 0.5 ml Eppendorf tubes using 40% acetonitrile, 0.1% formic acid. The eluted peptides were concentrated in an Eppendorf Speedvac and re-constituted in 50 mM HEPES (pH 8.5) for TMT labeling with 16plex tags (Thermo). A reference sample was prepared by mixing equal amounts of peptides from each sample and labeling them separately. Labeling was done according to the manufacturer’s instructions, and subsequently, labeled peptides were mixed 1:1:1:1:1:1:1:1:1:1:1, spiking in reference channel to each mix. TFA was added to acidify and bring acetonitrile concentration down to < 5%. Prior to mass spectrometry analysis, the peptides were desalted and fractionated using an offline ThermoFisher Ultimate3000 liquid chromatography system using high pH fractionation (5 mM Ammonium Bicarbonate, pH 10) at 5ul/min flowrate. 15ug of peptides were separated over a 120 min gradient (5% to 35% Acetonitrile), while collecting fractions every 130 s. The resulting 60 fractions were pooled into 30 final fractions, acidified to pH < 2 with 1% TFA and loaded onto EvoSep stagetips according to the manufacturer’s protocol.

### Mass Spectrometry Data Acquisition

For each fraction, peptides were analyzed using the pre-set “30 samples per day” method on the EvoSep One instrument. Peptides were eluted over a 44-min gradient and analyzed with an Orbitrap EclipseTM TribridTM instrument (Thermo Fisher Scientific) with FAIMS ProTM Interface (ThermoFisher Scientific) switched between CVs of − 50 V and − 70 V with cycle times of 1.5 s. Full MS spectra were collected at a resolution of 120,000, with a normalized automatic gain control (AGC) target set to “standard” or maximum injection time of 50 ms and a scan range of 375–1500 m/z. MS1 precursors with an intensity of > 5 × 103 and a charge state of 2–7 were selected for MS2 analysis. Dynamic exclusion was set to 60 s, the exclusion list was shared between CV values, and Advanced Peak Determination was set to “off.” The precursor fit threshold was set to 70% with a fit window of 0.7 m/z for MS2. Precursors selected for MS2 were isolated in the quadrupole with a 0.7 m/z window. Ions were collected for a maximum injection time of 50 ms, and the normalized AGC target was set to “standard.” Fragmentation was performed with a collision-induced dissociation (CID) normalized collision energy of 35%, and MS2 spectra were acquired in the IT at a scan rate rapid. The MS2 spectra were subjected to real-time search (RTS) using the reviewed Uniprot protein database Homo sapiens and trypsin set as an enzyme. Static modifications were TMTpro on lysine (K) and N-terminus and carbamidomethyl on cysteine (C). Oxidation of methionine (M) was set as variable modification. Maximum missed cleavages were set to 1 and maximum variable modifications to 2. FDR filtering was enabled, the maximum search time was set to 35 ms, and the scoring threshold was set to 1 Xcorr, 0 dCn, and 5 ppm precursor tolerance. Use as a trigger only was disabled and close-out was enabled with the maximum number of peptides per protein set to 4. Precursors were subsequently filtered with an isobaric tag loss exclusion of TMT and precursor mass exclusion set to 18 m/z low and 5 m/z high. Precursors identified by RTS were isolated for an MS3 scan using the quadrupole with a 2 m/z window, and ions were collected for a maximum injection time of 86 ms and normalized AGC target of 200%. Turbo TMT was deactivated, and the number of dependent scans was set to 5. Isolated precursors were fragmented again with 63% normalized higher-energy collisional dissociation (HCD) collision energy, and MS3 spectra were acquired in the orbitrap at 50000 resolution with a scan range of 100–500 m/z. MS performance was verified for consistency by running complex cell lysate quality control standard.

### Proteomics Normalization and Filtering

The raw files were analyzed using Proteome Discoverer 2.4 (Thermo Fisher Scientific). TMT reporter ion quantitation was enabled in the processing and consensus steps, and spectra were matched against the Homo sapiens database obtained from UniProt. Dynamic modifications were set as oxidation (M), and acetyl on protein N-termini. Cysteine carbamidomethyl (C) and TMT 16-plex (peptide N-termini and K) were set as static modifications. All results were filtered to a 1% FDR, and protein quantitation was done using the built-in Minora Feature Detector with statistical significance testing done with the built-in *t*-test. The peptide abundances are normalized based on the total peptide amount. Thereby, the total sum of identified peptides in a channel is normalized to the channel with the highest abundance. The protein or peptide abundances are then scaled to the NormMix channel (126) to form the same pool by scaling the NormMix channel to 100. All other channels are proportionally scaled up or down using the same factor.

Aggregated protein intensities from Proteome Discoverer were further processed in a downstream analysis using R. First, proteins labeled as “low” or “medium” for the protein FDR confidence were removed. Additionally, proteins for which more than 25% of the samples showed missing values were removed. The remaining missing values were imputed using a local least squares method implemented in the pcaMethods R package [56]. Using principal component analysis on the filtered and imputed dataset, we investigated batch effects. We observed that despite the batch correction described above, batch effects were still visible. In particular, samples from batch 6 were separated from the remaining samples along PC2 (Fig. S1). We thus decided to exclude these samples from the analysis.

### Cell Composition Estimation

Estimation of MGPs was performed as described [9] using cell-type markers from Kelley et al. [13] and Velmeshev et al.[14].

### Differential Expression Analysis

Before DPE, we performed surrogate variable (SV) analysis (R-package *sva* [57]) and explored correlations between first and second SV with possible covariates such as cell estimates, and age. In an iterative process, we added covariates to the base model and observed the remaining correlations. This was an exploratory process which is documented in the analysis code and helped us design the model.

DPE was performed using functions *lmFit* and *eBayes from* the *limma* R-package [58]. For all DPE analyses, we transformed the protein intensities to log scale. To test for differences between the disease groups and controls, we designed one model: “ ~ Age + Pool_factor (batch) + Sex + Neurons + Endothelials + Stratification,” where the stratification variable was transformed to 3 binary variables (PSP, PD, and MSA) indicating whether a sample belonged to the disease group or not. Similarly, the variable Pool_factor was transformed to multiple (*N* = 10) dummy variables by the “model.matrix” function, each indicating whether a sample came from the pool (batch) or not. In a second analysis, we tested for association between protein expression and Braak staging scores based on a sub-selection of samples. For this, we designed a separate model, where we did not include each binary pool variable in the model design. Due to the lower number of samples in this analysis and to reduce model complexity, we did the following. We performed a principal component analysis on the expression data of the selected samples. We observed a separation of samples belonging to different pools along the PC2. We tested this association with a linear model (PC2 ~ Pool_factor) and found they were significantly associated. We thus decided to include PC2 instead of multiple binary pool variables in the model design. The model was thus: “ ~ PC2 + neurons + endothelial + age + sex + Braak_LB.”

To test for geneset enrichment, we used the function multilevel_fgsea from the R package fgsea, version 1.21.45 [59]. Specific parameters are documented in the code for the analysis (see data access). For each score type, we ran the function on two genesets: (i) a simplified list of genesets from the Gene Ontology (GO) database and (ii) a list of genesets from the Kyoto Encyclopedia of Genes and Genomes (KEGG), accessed through MSigDB [60, 61]. Both lists are available as “.gmt” files in the code repository. To generate a simplified, non-redundant GO list, pathways from the complete GO databases (CC, BP, and MF) were clustered iteratively based on their similarity (Cohen’s kappa, κ) until no κ > 0.4. Geneset overrepresentation analysis was performed using WebGesatltR [62] and the geneset databases the package provides: “geneontology_Biological_Process” “geneontology_Biological_Process_noRedundant,” “geneontology_Cellular_Component,” “geneontology_Cellular_Component_noRedundant,” “geneontology_Molecular_Function,” “geneontology_Molecular_Function_noRedundant,” “pathway_KEGG” “pathway_Panther,” “pathway_Reactome,” “pathway_Wikipathway,” “pathway_Wikipathway_cancer,” “disease_Disgenet,” “disease_GLAD4U,” “disease_OMIM,” and “phenotype_Human_Phenotype_Ontology”. The pre-filtered set of proteins was used as a background.

Heatmaps in supplementary Figs. S3 and S4 were generated using Cytoscape [63]. The layout was manually arranged.

### Machine Learning Analysis

Differentially expressed proteins from the PD versus control analysis were divided into train and test datasets by randomly sampling 70% of the proteins into the train dataset and using the remaining as test dataset. The training dataset was used to tune a xgboost classifier using Gridsearch and *N* = 5 cross-validation. For this, we employed the R package “xgboost” [64]. The tuning was performed using the functions “trainControl” and “train” from the R package “caret” [65]. Xgboost parameters “eta,” “max_depth,” “gamma,” “colsample_bytree,” and “subsample” were tuned by optimizing the F1 score. For this, we employed the R package “MLmetrics” [66]. Variable importance was explored using the R package “treeshap” [67].

Our results are based on the best model according to this workflow. This model is provided as an R object in the code repository.

## Supplementary Information

Below is the link to the electronic supplementary material.

Supplementary file1 (DOCX 639 KB)

Supplementary file2 (XLSX 18 KB)

Supplementary file3 (XLSX 1091 KB)

Supplementary file4 (XLSX 12 KB)

## Data Availability

The datasets and code required to reproduce the results of these analyses are available at https://github.com/fifdick/DPE_parkinsonisms_brain. RAW Proteomics data are available for reviewers at https://www.synapse.org/#!Synapse:syn53644993.
