# Supplementary material for: Brain Proteome Profiling Reveals Common and Divergent Signatures in Parkinson’s Disease, Multiple System Atrophy, and Progressive Supranuclear Palsy
Source: Mol Neurobiol. 2024 Aug 21;62(3):2801–16. doi: 10.1007/s12035-024-04422-y (PMC11790761; doi:10.1007/s12035-024-04422-y)
Supplement: Supplementary file 1 — Supplementary file1 (DOCX 639 KB) [file 12035_2024_4422_MOESM1_ESM.docx]

**Supplementary material**


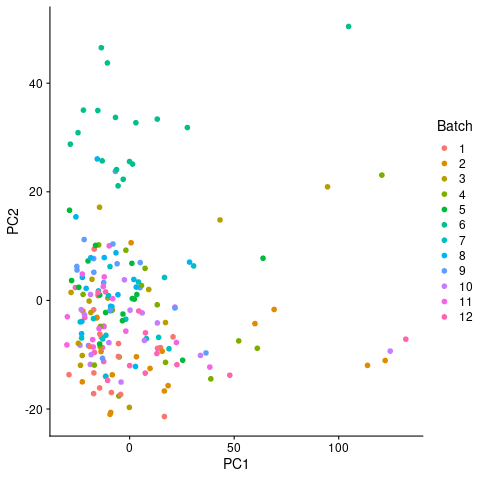


**Supplementary figure S1**

Principle component analysis of protein expression. Samples are displayed as datapoints in the first and second principle component space. Color indicates proteomics experiment batch (pool).


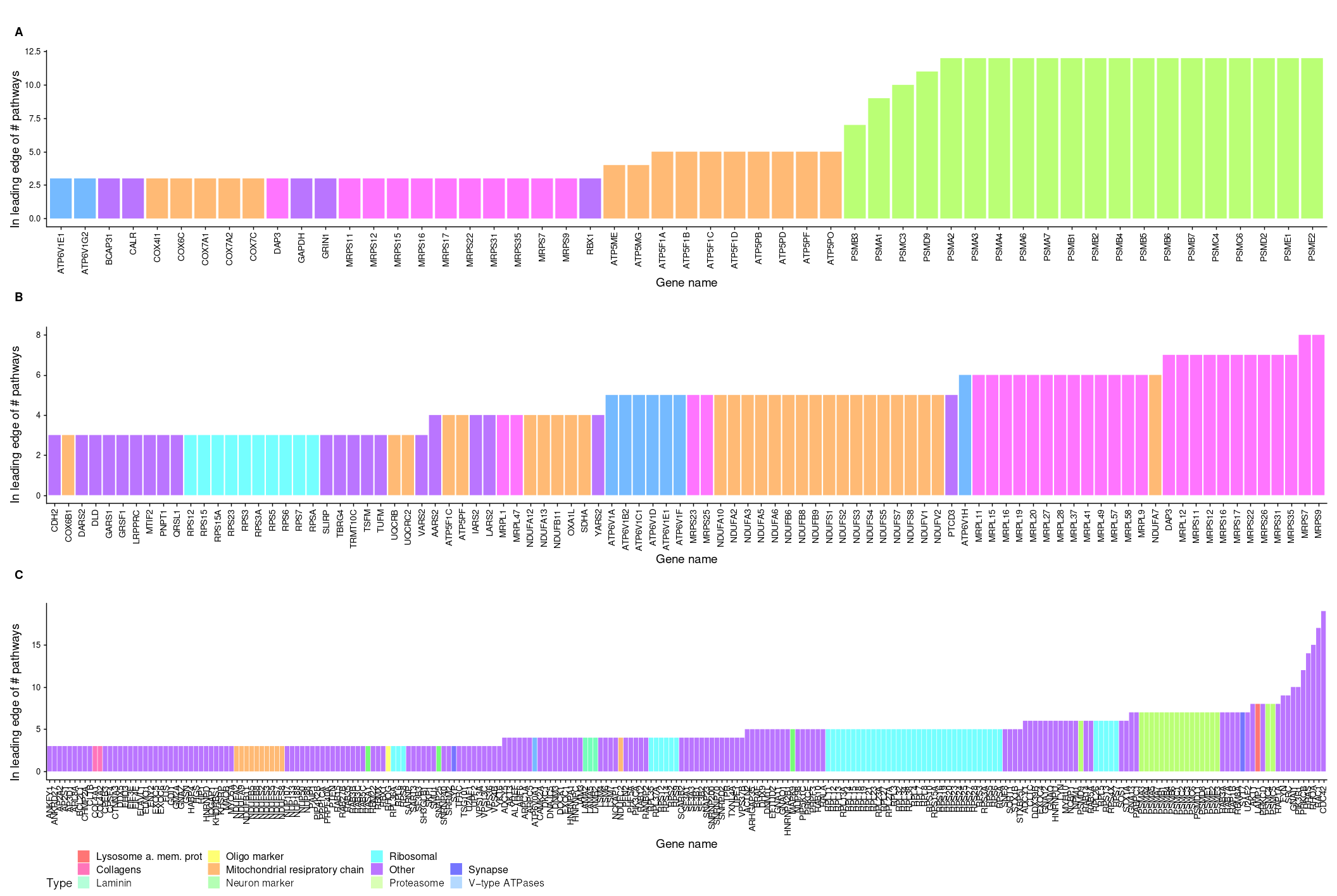


**Supplementary figure S2**

**Proteins driving pathway enrichment.**

Proteins which were part of at least two leading edges of significant pathways are sorted along the x-axis by number of leading edges they were part of (y-axis). Proteins were categorized manually according to their most prominent function reflected by color. Leading edge plots were generated for each pathway analysis in the comparison of **A:** PD versus Control **B:** PSP versus Control **C:** MSA versus Control.


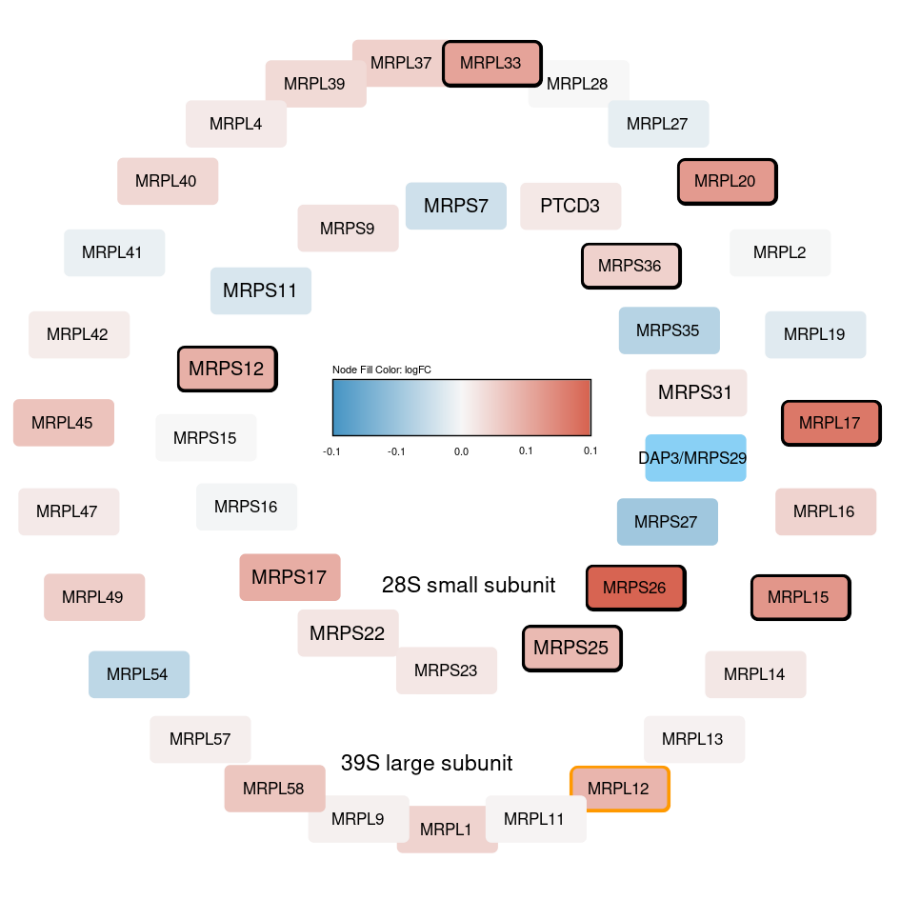

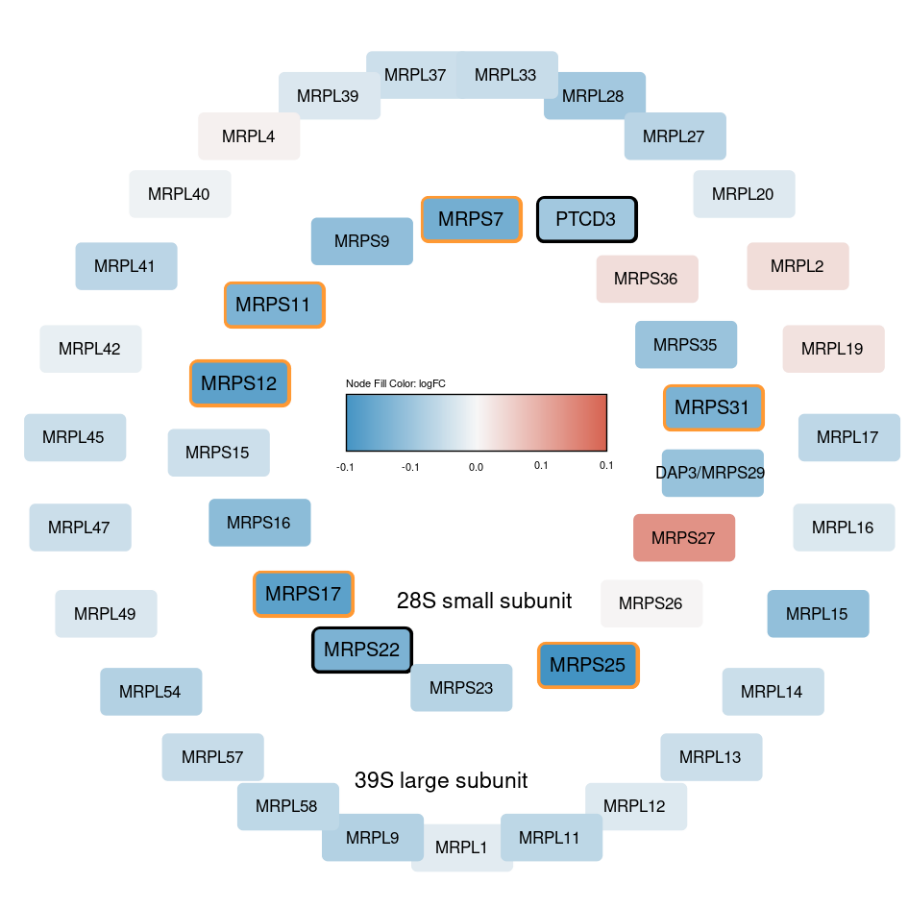


**A B**

**Supplementary figure S3**

**Fold change heatmap of mitochondrial ribosomal subunits**

Subunits of the mitochondrial ribosome are displayed as nodes. Node coloring indicates log fold change, node frame indicates nominal (black) and adjusted p-value (orange) significance from the analysis comparing A: PD versus Controls B: Braak alpha-synuclein within PDs.

**
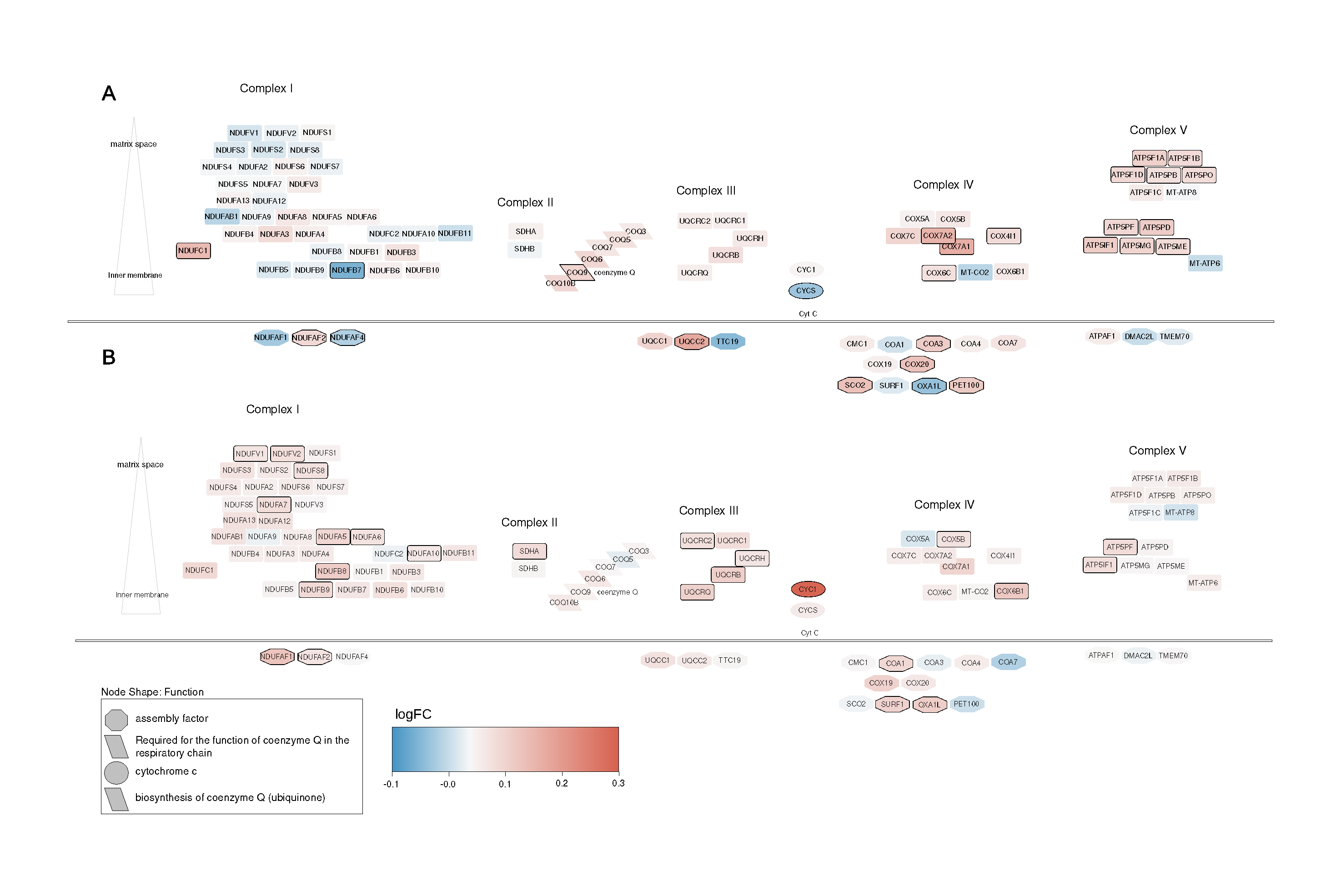
**

**Supplementary figure S4**

**Fold change heatmap of mitochondrial respiratory chain subunits**

Proteins of the mitochondrial respiratory chain are displayed as nodes. Node shape reflects protein function. Node coloring indicates log fold change, node frame indicates nominal significance (black) from the analysis comparing A: PD versus Controls B: Braak alpha-synuclein staging within PDs.

**Supplementary table S1**

Cohort demographics, including sex and age of all participants.

**Supplementary table S2**

All differentially expressed proteins identified in the differential protein expression (DPE) analysis.

**Supplementary table S3**

All proteins with a non-zero contribution to the separation of PD from controls in our model. The model was developed to predict if a patient belonged to the PD or control group by an ensemble learning methodology.
